# Sphincter preservation in distal CT2N0 rectal cancer after preoperative chemoradiotherapy

**DOI:** 10.1186/s13014-014-0233-3

**Published:** 2014-10-22

**Authors:** Nir Wasserberg, Yulia Kundel, Ofer Purim, Andrei Keidar, Hanoch Kashtan, Eran Sadot, Eyal Fenig, Baruch Brenner

**Affiliations:** Department of Surgery B, Petach Tikva, 49100 Israel; Davidoff Cancer Center, Rabin Medical Center, Beilinson Campus, Petach Tikva, 49100 Israel; Sackler Faculty of Medicine, Tel Aviv University, Tel Aviv, 69978 Israel

**Keywords:** T2 rectal cancer, Preoperative chemoradiotherapy, Sphincter preservation, Low anterior resection, Abdominoperineal resection

## Abstract

**Background:**

Preoperative chemoradiotherapy is usually not indicated for cT2N0 rectal cancer. Abdominoperineal resection is the standard treatment for distal rectal tumors. The aim of the study was to evaluate the actual sphincter-preservation rate in patients with distal cT2N0 rectal cancer given neoadjuvant chemoradiotherapy.

**Methods:**

Data were retrospectively collected for all patients who were diagnosed with distal cT2N0 rectal cancer at a tertiary medical center in 2000–2008 and received chemoradiotherapy followed by surgery (5–7 weeks later).

**Results:**

Thirty-three patients (22 male) of median age 65 years (range, 32–88) were identified. Tumor distance from the anal verge ranged from 0 to 5 cm. R0 resection with sphincter preservation was accomplished in 22 patients (66%), with a 22% pathological complete response rate. Median follow-up time was 62 months (range 7–120). There were no local failures. Crude disease-free and overall survival were 82% and 86%, respectively. Factors associated with sphincter preservation were tumor location (OR = 0.58, p =  0.02, 95% CI = 0.37-0.91) and pathological downstaging (OR = 7.8, p = 0.02, 95% CI = 1.35-45.85). Chemoradiotherapy was well tolerated.

**Conclusion:**

High rates of sphincter preservation can be achieved after preoperative chemoradiotherapy for distal cT2N0 rectal cancer, with tolerable toxicity, without compromising oncological outcome.

## Background

The main aims of rectal cancer treatment are loco-regional control and improvement of overall and disease-free survival. Currently, the standard treatment for rectal cancer consists of radical surgery with total or partial tumor-specific mesorectal excision. Short-course radiotherapy or long- course chemoradiotherapy (CRT) may be administered preoperatively, depending on the location of the tumor in the rectum and the disease stage [1]. For patients with very low-lying rectal tumors, abdominoperineal resection (APR) is the traditional method of choice [2]. However, the use of sphincter-sparing procedures has been increasing [3] owing to advances in surgical technique and instruments, the introduction of specialized high-volume centers, and an improved understanding of the sphincter mechanism and the tumor biology [1]. Consequently, sphincter-sparing surgery is now considered a desirable endpoint also in this patient group when it is technically, functionally, and oncologically feasible [4].

In terms of disease stage, preoperative CRT or radiotherapy is indicated for locally advanced cT3 and/or N + rectal tumors [5] to improve local control [6]. However, given its high toxicity, CRT is difficult to justify for patients with cT2N0 rectal cancer, in whom it has not been associated with considerable oncological improvement [7,8]. Nevertheless, CRT might be applicable in some cases in order to avoid APR when low anterior resection (LAR) is otherwise unfeasible.

There is some evidence that preoperative CRT, by downstaging and downsizing the tumor, may also benefit sphincter preservation [9,10]. Yet, because it is rarely used to treat early-stage disease, only sparse data are available on the actual rate of sphincter preservation in patients with distal cT2N0 rectal cancer who received CRT before surgery. In the only published trial that specifically addressed this issue, Rengan and colleagues [11] demonstrated that in patients with distal cT2N0 rectal cancer who require APR, preoperative pelvic irradiation improved sphincter preservation without any apparent cost to local control or survival. The aim of the present study was to evaluate the rate of sphincter preservation when preoperative CRT is used in an attempt to avoid APR in patients with distal cT2N0 rectal tumors.

## Methods

### Patients

The electronic database of a tertiary medical center was searched for all patients with rectal cancer treated by long-course CRT followed by curative surgical resection in 2000 to 2008. Among the 324 patients identified, we selected those with distal cT2N0 disease who were being clinically considered by their surgeons for an APR and were referred for preoperative CRT to increase the probability of a sphincter-preserving procedure. The diagnosis was confirmed on histological study in all cases, and staging was confirmed by thoracic and abdomino-pelvic computerized tomography, tocheck for distal metastasis, and endoscopic rectal ultrasound, for local evaluation.

### Treatment

Radiation was delivered with 6 or18 MV photons using a three-field belly-board technique. The superior border was placed at the L5-S1 interspace, and the inferior border was 3 to 4 cm distal to the tumor; in all cases, the inferior border was at or distal to the obturator foramen. In order to encompass the iliac lymph node chain, the lateral borders of the posterior portal were 1.5 cm outside the true bony pelvis. The lateral fields encompassed the sacrum and coccyx posteriorly and the femoral head anteriorly to include the obturator nodes.

The delineation of the planning target volume (PTV), gross total volume (GTV) and clinical target volume (CTV) was as follows. The whole pelvis (PTV1) including the primary tumor (GTV), internal, presacral, obturator, and perirectal lymph nodes (CTV1), received a total of 45 Gy, with the dose prescribed to the 95% isodose line using standard fractions of 1.8 Gy/day. This was followed by a reduced field boost (PTV2) delivered by the same technique (posterior and 2 lateral fields) to a smaller treatment volume that included the tumor (GTV) and a 2- to 3-cm margin for an additional 5.4 Gy.

Surgery was performed 5–7 weeks after completion of CRT. The type of surgery was left to the discretion of the operating surgeon. Postoperative management was based on the pathological staging.

### Data collection

For the present study, the following data were retrieved from the medical files of the eligible patients: demographics, pretreatment staging, findings on physical examination, rigid and flexible endoscopy, and local and systemic imaging, in addition to the toxicity assessment during preoperative treatment [according to the NCI (National Cancer Institute) Common Toxicity Criteria, Version 3 type of surgery, pathology results, and findings on follow-up.

The study was approved by the institutional review board.

### Statistical analysis

Outcome measures were rates of sphincter preservation, overall survival (OS), and disease-free survival (DFS) including local and distant recurrence. Univariate logistic regression was applied to analyze putative predictors of sphincter preservation. Significance was defined as p <0.05. OS was calculated from the date of surgery to death or the last date the patient was known to be alive. DFS was calculated from the date of surgery to the first documented recurrence or the last date the patient was known to be free of recurrence. OS and DFS were estimated using the Kaplan-Meier method and compared between patients who underwent LAR or APR using the log-rank test.

## Results

### Patients

Thirty-three patients were eligible for inclusion in the study: 22 (66%) male and 11 female of median age 65 years (range 32–88). Distance of the tumor from the anal verge ranged from 0 to 5 cm on rigid rectoscopy. Twenty-two patients (66%) underwent LAR with sphincter preservation and 11 underwent APR. All patients had an R0 resection with clear distal and circumferential margins. The tumor was downstaged after CRT in 14 patients (42%), of whom 7 (22% of the entire group) had pT1 and 7 (22% of the entire group) had a pathological complete response (pCR). There were no significant differences in tumor charecteristics between tumors with or without pathological downstaging (Table 1). Advanced pathological stage pT3 was found in 2 patients overall, and positive N1 was found in one patient. Table 2 describes the preoperative patient and tumor characteristics by type of surgery performed, and Table 3 describes the postoperative results.

**Table 1:** **Tumor characteristics in 33 patients by pathological downstaging vs. no pathological downstaging**

**No pathological downstaging (n = 19)**	**Pathological downstaging (n = 14)**	**Characteristics**
Pre-CRT serum CEA level (mcg/L), mean ± SD	5.4 ± 4.7	15.9 ± 40.7
Tumor diameter (cm), median (range)	4 (2–12)	5 (1–13)
Tumor fixation, %	2	2
% circumference, median (range)	50 (30–100)	60 (30–100)
Tumor grade, n (%)		2 (10)
1	4 (28)	13 (68)
2	7 (50)	4 (21)
3	3 (21)	
Mucin, n	1	2
Lymphovascular or perineural invasion, n	2	

CRT - chemoradiotherapy, CEA - carcinoembryonic antigen.

**Table 2:** **Clinical characteristics in 33 patients with distal cT2N0 rectal cancer by type of surgery**

**Characteristics**	**APR (n = 11)**	**LAR (n = 22)**
Age (yr), median (range)	67 (60–77)	64 (32–88)
Gender, n (%)		
Male	7 (63)	15 (68)
Female	4 (37)	7 (32)
Clinical staging, n (%)		
cT2	11 (100)	22 (100)
cN0	11 (100)	22 (100)
Tumor diameter (cm), median (range)	5 (3–8)	5 (2–13)
% circumference, median (range)	45 (30–100)	60 (30–100)
Tumor fixation, n (%)	2(18)	2(9)
Tumor grade, n (%)		
1	1(9)	5(22)
2	7(63)	13(59)
3	3(27)	4(18)
Mucin, n (%)	0(0)	1(4)
Distance from anal verge (cm),		
Median (range)	3 (0–5)	4 (0–5)

APR-abdominoperineal resection, LAR-low anterior resection.

**Table 3:** **Postoperative results in 33 patients with distal cT2N0 rectal cancer by type of surgery**

**Characteristics**	**APR(n = 11)**	**LAR (n = 22)**	**Total(n = 33)**
R0 resection *	11	22	33
pT3	2	0	2 (6%)
Pathological downstaging	2	12	14 (42%)
pT1	1	6	7 (22%)
pCR	1	6	7 (22%)
Tumor grade			
1	2	5	7
2	5	13	18
3	4	4	8
Mucin	3	0	3
Lymphovascular or perineural invasion	2	1	3
Number of retrieved lymph nodes, median (range)	6 (3–16)	5 (3–11)	8 (4–17)
pN1	1	0	1

*Proximal, distal, and radial margins.

APR-abdominoperineal resection, LAR-low anterior resection.

Values are n or n(%) unless otherwise indicated.

### Predictors of sphincter preservation

Factors associated with sphincter preservation (Table 4) were tumor distance from the anal verge (OR = 0.58, p = 0.02, 95% CI = 0.37-0.91 for every centimeter distance) and pathological downstaging including pCR (OR = 7.8, p = 0.02, 95% CI = 1.35-45.85). There was no association with patient age, sex or tumor size, fixation, percentage circumference, or preoperative ultrasound staging *Long-term outcome.*

**Table 4:** **Factors associated with sphincter preservation (univariate analysis)**

**Factor**	**OR**	**P value**	**95% CI**
Distance from anal verge	0.58	0.02	0.37–0.91
Age	1	0.2	0.9–1.1
Sex	1.2	0.9	0.26–5.6
Tumor size	0.8	0.4	0.6–1.2
% circumference	0.9	0.6	0.5–1.7
uT downstaging	3.9	1.45	0.6–25
pT downstaging	7.8	0.02	1.35-45.85

pT-pathological tumor, uT, ultrasound observation of T stage.

**Figure 1:**
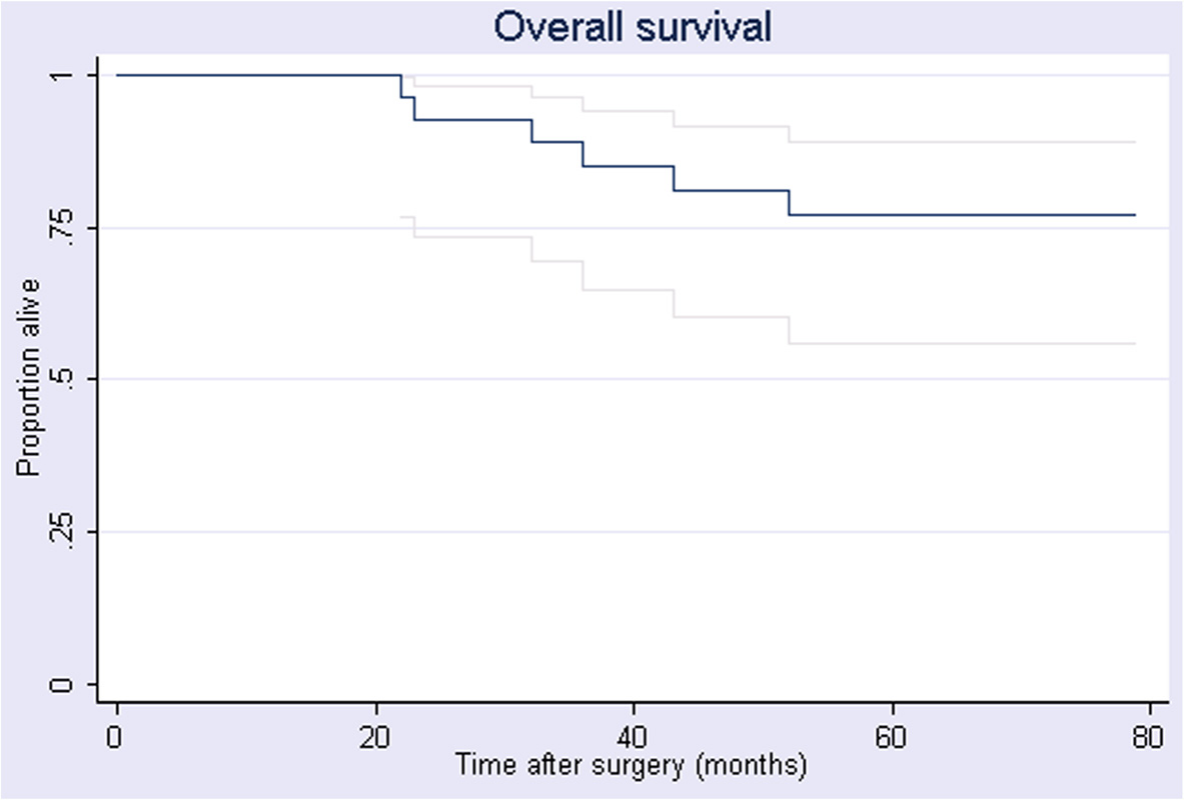
**Kaplan-Meier estimated 5-year overall survival in 33 patients with cT2N0 rectal cancer treated with CRT followed by surgery.**

**Figure 2:**
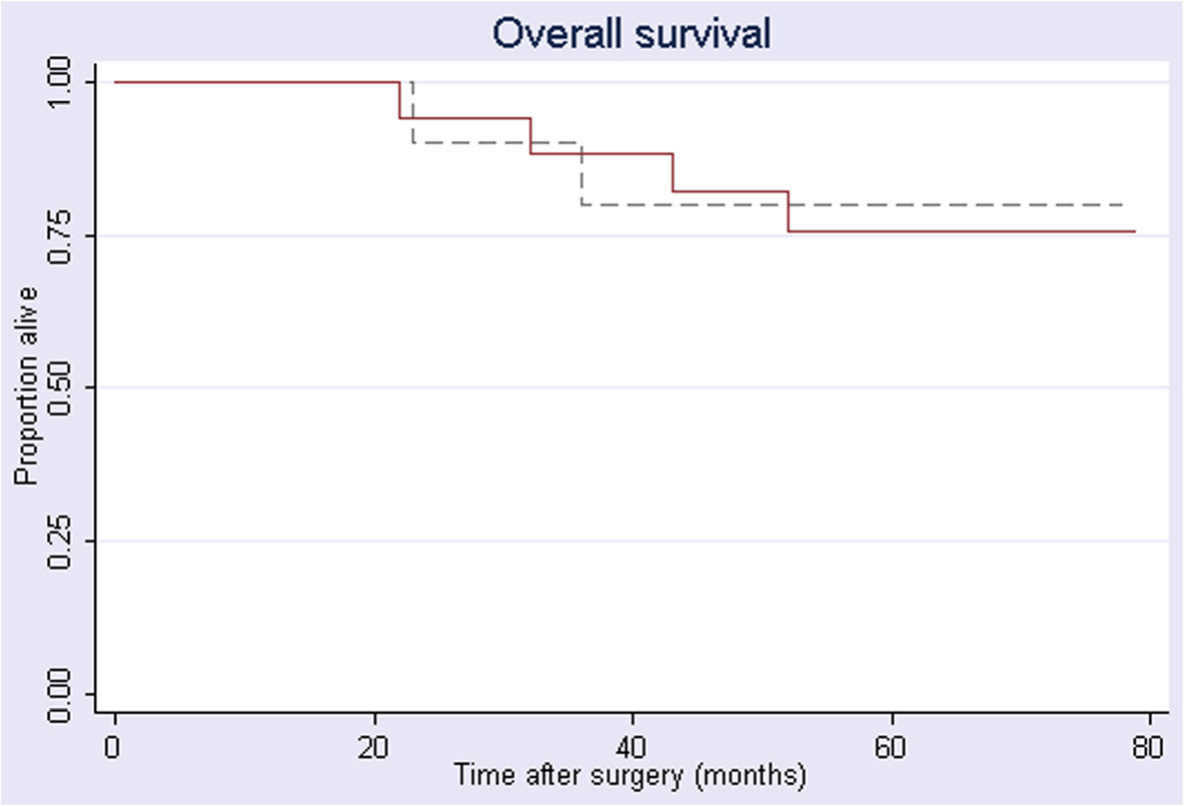
**Kaplan-Meier estimated 5-year overall survival in 33 patients with cT2N0 rectal cancer treated with CRT followed by surgery, by type of surgery.**

The median duration of follow-up was 62 months (range, 7–112 months): 54 months for the LAR group (range, 7–112 months) and 89 months for the APR group (range, 23–110 months). There were no local failures in either group. Distant recurrence was documented in 3 patients in the APR group (27%) and 2 in the LAR group (9%). The estimated 5-year OS was 77% for the entire cohort (Figure 1), 80% for the APR group, and 75% for the LAR group (Figure 2). DFS for the whole cohort was 76%; the estimated rate was 70% for the APR group and 80% for the LAR group. There was no statistically significant between-group difference in either OS or DFS.

### CRT toxicity

CRT was well tolerated (Table 5). Grade 3 thrombocytopenia was documented in 13% of patients, but there were no other cases of severe toxicity and no treatment-related admissions. The most common side effect was grade 1–2 dermal reaction, in 30% of patients. A low-grade gastrointestinal reaction occurred in less than 20% of patients, and only one patient complained of severe anal pain. There were no treatment-related deaths.

**Table 5:** **Toxic effect of CRT in 33 patients with distal cT2N0 rectal cancer**

**Toxicity**	**Grade 1-2**		**Grade 3-4**	
	**Number**	**%**	**Number**	**%**
Hematological				
Neutropenia	2	6	0	0
Neutropenic fever	0	0	0	0
Anemia	3	9	0	0
Thrombocytopenia	0	0	3	13.0
Bleeding	1	3	0	0
Nonhematological				
Nausea	1	3	0	0
Vomiting	0	0	0	0
Fatigue	0	0	0	0
Diarrhea	4	10	0	0
Stomatitis	1	3	0	0
Dermal	10	30	0	0
Anorexia	0	0	0	0
Proctitis	5	15	0	0
Anal pain	4	12	1	3

## Discussion

Efforts to preserve the sphincter in rectal cancer surgery are based on oncological, anatomical, and technical considerations in addition to the patient’s co-morbid status and his or her desire. The present study indicates that preoperative CRT may benefit patients with distal cT2N0 tumors in terms of an increased rate of sphincter preservation without compromising the oncological outcome.

Our finding supports earlier claims of an improvement in sphincter preservation after preoperative CRT [9,12]. However, the issue remains controversial. The German Rectal Cancer Study Group [6] found that preoperative CRT was associated with a significantly higher incidence of sphincter preservation than postoperative CRT in patients clinically considered for APR before treatment, but there was no difference in the overall rate of sphincter preservation between the groups. The body of evidence from randomized clinical trials [13-15] did not support the benefit of preoperative CRT for sphincter preservation, although in the Lyon R90-01 trial [13], a trend was noted toward more conservative surgery, especially in low rectal tumors (41% vs.23%), after a longer interval between CRT and surgery. Specific data for patients with low cT2 rectal cancer are limited. Some reports on mixed populations including patients with cT2 tumors [14,16] noted high rates of sphincter preservation after preoperative CRT. In the study of Rengan et al. [11], of 27 patients with cT2N0 distal rectal tumors who received preoperative CRT, 78% underwent sphincter-sparing surgery, with a 5-year OS of 86% and a 5-year DFS of 77%. These results are comparable to the present study in which sphincter-preserving surgery was feasible in 66% of patients after CRT.

Factors influencing the feasibility of sphincter preservation in our study were tumor distance from the anal verge and tumor downstaging. The majority of patients in our cohort were considered clinically by their surgeons for an APR in relation to the distance of the tumor from the anal verge. Other objective parameters that may have supported this decision were unavailable to us owing to the retrospective nature of the study. The distance from the anal verge is commonly used to describe the tumor location and to assist in decision-making, although it is less accurate than an anatomical description [17]. In a large observational study that evaluated factors predicting sphincter preservation in patients with rectal cancer treated at institutions of the National Comprehensive Cancer Network [18], sphincter-preserving surgery was found to be more likely if the tumor was located >6 cm from the anal verge (OR = 7.7). Other predictive factors included younger age at diagnosis, nonfixed tumor, and the operating institution [18]. Pathological tumor downstaging may also reflect tumor downsizing. Indeed, in the present study, the number of pCRs was higher in the LAR than the APR group. Several studies reported a correlation between response to preoperative treatment and sphincter preservation [18-22]. Although all patients in the present study underwent radical excision, the high rates of clinical complete response (with subsequent pCR) in low T2 rectal cancer should also be considered when weighing the possibility of organ preserving treatment in selected patients [22]. Although low pre-CRT carcinoembryonic levels were shown in an earlier study to be associated with tumor response to CRT [22], we found no significant differences in carcinoembryonic levels or other relevant parameters between tumors with or without downstaging.

Preoperative CRT poses a risk of some adverse short- and long-term consequences, including radiation enteritis, diarrhea, ileus, hematologic toxicities and functional anorectal and genitourinary impairments [6,23,24]. Although CRT is not recommended for cT2N0 rectal cancer [25], in the present study, it was very well tolerated, with very few, mostly mild treatment-related side effects. This was also reported by Rengan et al. [11].

This study was limited by its retrospective nature and small sample size. Nevertheless, it includes one of the largest cohorts with cT2NO rectal cancer treated with neoadjuvant CRT to date. The retrospective design made it difficult to assess postoperative function. The functional data were retrieved from follow-up charts and showed mild impairment postoperatively in the LAR group, yet none of the patients reported complete incontinence. Additionally, we were unable to compare the groups for postoperative quality of life. Impaired functional outcomes for LAR with preoperative CRT have been well described in the literature [26,27]. It appears that patients undergoing sphincter-preserving surgery for rectal cancer have some degree of impaired bowel function, and those treated with radiation, coloanal anastomoses, or hand-sewn anastomoses have significantly worse function regardless of the preoperative tumor stage [11].

In our study, a median number of 8 lymph nodes were retrieved (range 4–17); several studies recommend examination of at least 12 lymph nodes in the colorectal specimen. This number is related to patient prognosis and is used to reflect surgical quality [28,29]. Studies that focused on this issue in rectal tumors showed that after long-term CRT, there was a continuous decrease in the number of examined lymph nodes compared to non-irradiated specimens, with the mean number of detected nodes ranging between 4 and 14 per specimen [30,31]. A decrease in the number of retrieved nodes does not represent an inferior oncological outcome [32].

## Conclusion

The current standard treatment for patients with low cT2N0 rectal cancer is APR if an LAR is technically impossible or oncologically unsafe. Our results indicate that preoperative CRT may increase the rate of sphincter preservation without compromising the oncological outcome, with a relatively low rate of treatment-related adverse events. In the current era of multiple therapy options and increased patient participation, neoadjuvant CRT for patients with low lying cT2N0 rectal cancer may serve to reduce APR rates in these patient.

## Abbreviations

APR: Abdominoperineal resection
CRT: Chemoradiotherapy
CTV: Clinical target volume
DFS: Disease-free survival
GTV: Gross tumor volume
LAR: Low anterior resection
OS: Overall survival
pCR: Pathological complete response
PTV: Planning target volume
